# Toll-like receptor 7 protects against intestinal inflammation and restricts the development of colonic tissue-resident memory CD8^+^ T cells

**DOI:** 10.3389/fimmu.2024.1465175

**Published:** 2024-10-11

**Authors:** Hussein Hamade, Masato Tsuda, Naoki Oshima, Dalton T. Stamps, Michelle H. Wong, Jasmine T. Stamps, Lisa S. Thomas, Brenda C. Salumbides, Caroline Jin, Jordan S. Nunnelee, Deepti Dhall, Stephan R. Targan, Kathrin S. Michelsen

**Affiliations:** ^1^ F. Widjaja Inflammatory Bowel Disease Institute, Department of Medicine, Cedars-Sinai Medical Center, Los Angeles, CA, United States; ^2^ Department of Pathology and Laboratory Medicine, Cedars-Sinai Medical Center, Los Angeles, CA, United States; ^3^ Department of Biomedical Sciences, Cedars-Sinai Medical Center, Los Angeles, CA, United States

**Keywords:** TLR7, ATG16L1, CD8+ Trm, inflammatory bowel diseases, autophagy, plasmacytoid dendritic cells

## Abstract

**Introduction:**

The maintenance of intestinal homeostasis depends on a complex interaction between the immune system, intestinal epithelial barrier, and microbiota. Alteration in one of these components could lead to the development of inflammatory bowel diseases (IBD). Variants within the autophagy gene *ATG16L1* have been implicated in susceptibility and severity of Crohn’s disease (CD). Individuals carrying the risk *ATG16L1* T300A variant have higher caspase 3-dependent degradation of ATG16L1 resulting in impaired autophagy and increased cellular stress. ATG16L1-deficiency induces enhanced IL-1β secretion in dendritic cells in response to bacterial infection. Infection of ATG16L1-deficient mice with a persistent strain of murine norovirus renders these mice highly susceptible to dextran sulfate sodium colitis. Moreover, persistent norovirus infection leads to intestinal virus specific CD8^+^ T cells responses. Both Toll-like receptor 7 (TLR7), which recognizes single-stranded RNA viruses, and ATG16L1, which facilitates the delivery of viral nucleic acids to the autolysosome endosome, are required for anti-viral immune responses.

**Results and discussion:**

However, the role of the enteric virome in IBD is still poorly understood. Here, we investigate the role of TLR7 and ATG16L1 in intestinal homeostasis and inflammation. At steady state, *Tlr7^-/-^* mice have a significant increase in large intestinal lamina propria (LP) granzyme B^+^ tissue-resident memory CD8^+^ T (T_RM_) cells compared to WT mice, reminiscent of persistent norovirus infection. Deletion of *Atg16l1* in myeloid (*Atg16l1^ΔLyz2^*) or dendritic cells (*Atg16l1^ΔCd11c^*) leads to a similar increase of LP T_RM_. Furthermore, *Tlr7^-/-^* and *Atg16l1^ΔCd11c^* mice were more susceptible to dextran sulfate sodium colitis with an increase in disease activity index, histoscore, and increased secretion of IFN-γ and TNF-α. Treatment of *Atg16l1^ΔCd11c^* mice with the TLR7 agonist Imiquimod attenuated colonic inflammation in these mice. Our data demonstrate that ATG16L1-deficiency in myeloid and dendritic cells leads to an increase in LP T_RM_ and consequently to increased susceptibility to colitis by impairing the recognition of enteric viruses by TLR7.

**Conclusion:**

In conclusion, the convergence of ATG16L1 and TLR7 signaling pathways plays an important role in the immune response to intestinal viruses. Our data suggest that activation of the TLR7 signaling pathway could be an attractive therapeutic target for CD patients with *ATG16L1* risk variants.

## Introduction

Inflammatory bowel diseases (IBD) are heterogeneous chronic inflammatory disorders caused by dysregulated immune responses to intestinal microbiota in genetically predisposed individuals (1, 2). The two major types of IBD are Crohn’s disease (CD), which can affect any segment of the gastrointestinal tract, and ulcerative colitis (UC), which is limited to the colonic mucosa. Genome-wide association studies have identified over 260 loci involved in immune responses, host-microbial interactions, and cellular processes such as autophagy to be associated with susceptibility to IBD (1, 3–6). Variants within the autophagy gene *ATG16L1* have been implicated in the severity of CD (7, 8). Individuals carrying the risk *ATG16L1* T300A variant have higher caspase 3-dependent degradation of ATG16L1 resulting in impaired autophagy and increased cellular stress, Paneth cells abnormalities, and exhibit a higher incidence of CD (9–12). A knock-in mouse model expressing *Atg16L1* T300A does not develop spontaneous inflammation, but exhibit altered cytokine signaling and decreased antibacterial defense (13). *Atg16l1^HM^* mice, an engineered mouse strain with low expression of ATG16L1, infected with a persistent strain of murine norovirus (MNV), demonstrated aberrant secretion of Paneth cell granules strongly resembled those observed in individuals carrying the *ATG16L1* T300A variant and higher susceptibility to DSS-induced colitis compared to WT mice (14). Intriguingly, this effect was absent in mice infected with a non-persistent MNV strain or with inactivated MNV (14). In response to viral infections innate immune cells activate the autophagy pathway leading to degradation and elimination of invading viruses (15).

Infections with single-stranded RNA viruses are detected by the endosomal pattern recognition receptors TLR7 and TLR8 (16). Upon binding to their ligands, TLR7 recruits a Toll–IL-1 receptor (TIR) domain-containing adaptor MyD88, initiating the activation of the MyD88-dependent pathway (17). This cascade leads to the induction of type 1 interferons, and TNF-α, IL-6, IL-1β, and IL-12 (18–20). TLR7 is mainly expressed by plasmacytoid dendritic cells (pDCs) which produce copious amounts of type I IFNs during viral infection (21–25). PDCs deficient in the autophagy protein 5 (ATG5) exhibit diminished TLR7-dependent IFN-α and IL-12 p40 production when infected with vesicular stomatitis virus (VSV) or Sendai virus (SeV), suggesting that autophagy and formation of autophagosomes and viral degradation upstream of TLR7 are required for TLR7 activation (26). During later stages of infection, autophagy also facilitates antigen processing and thereby the induction of adaptive immune responses (27, 28). Interestingly, comprehensive profiling of immune responses to eukaryotic viruses revealed that infection with the persistent MNV strain CR6 induces an increase in granzyme B^+^ CD8^+^ T cells in the colonic LP, whereas the non-persistent strains CW3 did not (29).

Tissue-resident memory CD8^+^ T cells (T_RM_) are a specialized subset of non-circulating memory T cells that serve as a crucial first line of defense against reinfections in peripheral non-lymphoid tissues, including the intestinal mucosa (30, 31). T_RM_ cells are characterized by a unique transcriptional signature and are commonly identified by low expression of CD62L and high expression of CD69 and CD103 (32–35). CD69^+^CD103^+^ T_RM_ cells (both CD4^+^ and CD8^+^) were shown to be increased in the colon of patients with UC and CD and high levels of CD4^+^ T_RM_ cells in IBD patients were associated with early relapse (36). Mucosal CD4^+^ T_RM_ cells have been shown to produce copious amounts of IFN-γ, IL-2 and are the major source of TNF-α and IL-17 in CD patients (37–39). Additionally, CD8^+^ T_RM_ cells produce substantial quantities of granzyme B, granzyme K, and perforin, essential for executing cytotoxic functions, and IL-26 in UC, suggesting a role for CD8^+^ T_RM_ cells in the pathogenesis of UC (37, 40–42).

In this study, we investigate the role of TLR7 signaling pathway and its convergence with ATG16L1 pathway in intestinal homeostasis and inflammation. Our findings reveal that TLR7-deficiency promotes the development of LP CD8^+^ T_RM_ cells under steady-state condition and exacerbates susceptibility to dextran sulfate sodium (DSS)-induced colitis. *Tlr7^-/-^* mice developed more severe colonic inflammation, had an increased disease activity index, increased secretion of IFN-γ and TNF-α, and increased abundance of LP CD8^+^ T_RM_ cells. Similarly, to the phenotype we observed in *Tlr7^-/-^* mice, myeloid- and DC-specific deletion of ATG16L1 (*Atg16l1^ΔLyz2^* and *Atg16l1^ΔCd11c^* mice) resulted also in elevated LP CD8^+^ T_RM_ cells and increased susceptibility to DSS colitis. Since ATG16L1 is crucial in facilitating the delivery of viral nucleic acids to the endosomes where they activate TLR7 signaling, we hypothesized that treatment with the TLR7 agonist Imiquimod could bypass the requirement of ATG16L1 resulting in ameliorated colonic inflammation. *Atg16l1^ΔCd11c^* mice treated with Imiquimod had attenuated DSS-induced inflammation compared to untreated mice with significantly decreased disease activity index, and abundance of LP CD8^+^ T_RM_ cells. Consequently, our data demonstrate that ATG16L1-deficiency in myeloid cells promotes LP T_RM_ cell accumulation and increases the susceptibility to colitis by impairing the activation of TLR7 signaling in response to RNA viruses. Our findings suggest an important convergence of the ATG16L1 and TLR7 signaling pathways in myeloid cell-mediated immune responses to intestinal viruses and protection against the development of colitis.

## Materials and methods

### Mice

C57BL/6J, C57BL/6 Rag1^-/-^, CD11c-Cre, Lyz2-Cre mice, and *Tlr7^-/-^* mice (stock # 008380) (43) were purchased from the Jackson Laboratory (Bar Harbor, ME). The generation of *Atg16l1^f/f^* mice was previously described (44). To generate mice with conditional deletion of Atg16l1 specifically in myeloid or DCs, *Atg16l1^f/f^* mice were bred with transgenic mice expressing the *Cre* recombinase gene under the control of the *Lyz2 or Cd11c* promoter, Lyz2-Cre and CD11c-Cre mice, respectively. Female mice were used for both chronic and acute DSS-induced colitis experiments, while male mice were used for T cell transfer experiments. Both female and male mice were utilized in steady-state experiments. Mice were maintained under specific pathogen-free conditions in the Animal Care Facility at Cedars-Sinai Medical Center. Murine norovirus (MNV) was detected in feces of all experimental mice using a protocol described previously (45). All mice used in experiments were handled according to the guidelines and approved protocols of the Cedars-Sinai Medical Center Animal Care and Use Committees (IACUC protocol # 9214).

### Induction and assessment of acute and chronic colitis

Acute colitis was induced in female mice with 3% (w/v) DSS drinking water ad libitum for 7 days followed by one day of regular drinking water before being euthanized. Mice were checked daily for development of colitis by monitoring body weight, gross rectal bleeding, and stool consistency. Mice were sacrificed at day 8. Chronic colitis was induced by multi-cycle administration of DSS drinking water (46). Female mice of 8 weeks of age received 2.8% (w/v) DSS drinking water (40,000–50,000 MW) (MP Biomedicals, Irvine, CA) water on days 1-5, 8-12, 15-19, and 22-26. Mice were sacrificed at day 29. The MLN, cecum, colon, and rectum were removed. Tissues were fixed in 10% Formalin buffered phosphate, paraffin-embedded, and cross sections were stained with hematoxylin and eosin (H&E). H & E sections of cecum, colon, and rectum were scored by a trained observer blinded to the genotypes and treatments as described previously for acute DSS (47) or chronic DSS (46).

### T cell transfer model

Female C57BL/6 and *Tlr7^-/-^* mice were used for donors. Male *Rag1^-/-^* mice of the C57BL/6 background were used for recipients. Splenic CD4^+^ T cells were negatively selected using the EasySep Mouse CD4^+^ T Cell Enrichment Kit (STEMCELL Technologies Inc., Vancouver, Canada). Cells were labeled with anti-CD4, anti-CD25, and anti-CD45RB. Using the MoFlow cell sorter (Dako Cytomation, Carpinteria, CA), CD4^+^ CD25^-^ CD45RB^high^ cells were purified by gating and sorting 40% of the highest fluorescing CD45RB cells. Each recipient mouse was injected i.p. with 0.5 x 10^6^ cells in sterile PBS. Mice were weighed and observed for signs of colitis over a 6–8-week period. Mice were sacrificed at the indicated time points. Cecum, colon, and rectum were collected, formalin-fixed, paraffin-embedded, and stained with hematoxylin and eosin (H&E). Intestinal inflammation was scored by a semi-quantitative scoring system by a trained pathologist blinded to the experimental conditions (48). Single-cell suspensions of spleens, MLN, or lamina propria mononuclear cells (LPMC) were stained with anti-CD4 antibodies. For intracellular staining, cells were re-stimulated with 50 ng/ml PMA and 500 ng/ml ionomycin in the presence of Monensin for 4 h. Cells were stained anti-CD4 antibodies, fixed, permeabilized with Foxp3 staining buffer set (eBioscience), and intracellular stained with antibodies against IFN-γ ( XMG1.2) and IL-17 (eBio17B7) and analyzed using LSR II analyzer (BD Biosciences). Cells were restimulated with anti-CD3ε/anti-CD28 antibodies for 3 days. Cytokine levels in supernatants were measured by ELISA.

### ELISA

Cytokine concentration in culture supernatants was assayed by ELISA for IFN-γ, TNF-α, IL-17A, IL-22, and IL-12p40 (eBioscience, San Diego, CA).

### Quantitative RT-PCR

Total RNA was isolated using RNeasy kits and reverse transcribed into cDNA with Omniscript RT kit (both Qiagen). QPCR was performed using the CFX Opus real-time PCR system (Bio-Rad). SsoAdvanced Universal SYBR^®^ Green Supermix (Bio-Rad) was used. mRNA expression of *Ifnb1* was normalized to the expression of *Actb*. The relative gene expression was calculated by the 2^-^ΔΔ^Ct^ method.

### Isolation of mononuclear cells from LP and flow cytometry analysis

LPMC were isolated from the large intestine as previously described (46, 49). Mesenteric lymph nodes (MLN) were removed from the peritoneal cavity and mesenteric fat was removed. Single cell suspension was isolated by crushing MLN between two glass slides and passing the cell suspension through a 40 µm cell strainer. We routinely recovered LPMC at a viability of 76 - 91% with similar viabilities between genotypes. Single cell suspensions were stained with antibodies against murine CD11c (N418), CD103 (2E7), MHC-II (M5/114.15.2), TCRβ (H57-597), CD8β (H35-17.2), CD62L (MEL-14), CD69 (H1.2F3), and granzyme B (GB11), PD-1 (RMP1-30), CD4 (RM4-5) (all eBioscience). The following gating strategy was used to analyze LPMC and MLN: immune cells were gated based on forward and side scatter excluding cell aggregates. CD8^+^ T_RM_ subsets were gated first on MHCII^-^CD11c^+^CD103^+^ and then on TCRβ^+^ CD8β^+^. CD8^+^ T_RM_ were identified as CD11c^+^MHCII^-^CD103^+^TCRβ^+^CD8β^+^. For intracellular staining, cells were re-stimulated with 50 ng/ml PMA and 500 ng/ml ionomycin in the presence of Monensin for 4 h. Cells were stained with CD8β^+^ or CD4^+^, fixed, permeabilized with Foxp3 staining buffer set (eBioscience) and intracellular stained with antibodies against Granzyme B, IFN-γ, and IL-17 (eBioscience), and analyzed using a flow cytometer. Samples were acquired by flow cytometry using a LSR II analyzer (BD Biosciences) and analyzed using FlowJo software (TreeStar Inc).

### Bone-marrow derived plasmacytoid DCs

Bone-marrow (BM) cells were isolated by flushing femurs with RPMI-1640 medium supplemented with 10% FBS, 50 µg/ml gentamicin, 0.25 µg/ml amphotericin B, 100 U/ml Penicillin G, 0.1 mg/ml Streptomycin, and 50 µM β-mercaptoethanol (Complete RPMI-1640). BM cells were washed twice and cultured for 8 days with complete RPMI-1640 supplemented with 30 ng/ml murine Flt3L to stimulate differentiation to pDCs. After 8 days, cells were harvested, washed, and enriched for pDCs using EasySep™ Mouse plasmacytoid DC isolation kit (Catalog #19724). Enriched pDCs were stimulated for 24 hours with 100 ng/ml LPS or 5µg/ml Imiquimod.

### *In vivo* imiquimod treatment

Mice received a preventive treatment of Imiquimod before and during the DSS administration. Imiquimod (R837, InvivioGen) was resuspend in sterile PBS and 300 µg/mouse were i.p. injected 3 days before (D-3), and day 1, 4, and 7 of DSS treatment. Mice were monitored daily for development of colitis by assessing body weight, gross rectal bleeding, and stool consistency.

### Statistical analysis

Data were analyzed using GraphPad Prism software and presented as means ± SD. Differences between two groups were analyzed using the unpaired, 2-tailed Student *t* test. Differences were considered significant at *p* < 0.05.

## Results

### TLR7-deficiency leads to increased development of CD8^+^ T_RM_ in the intestine

**Figure 1:**
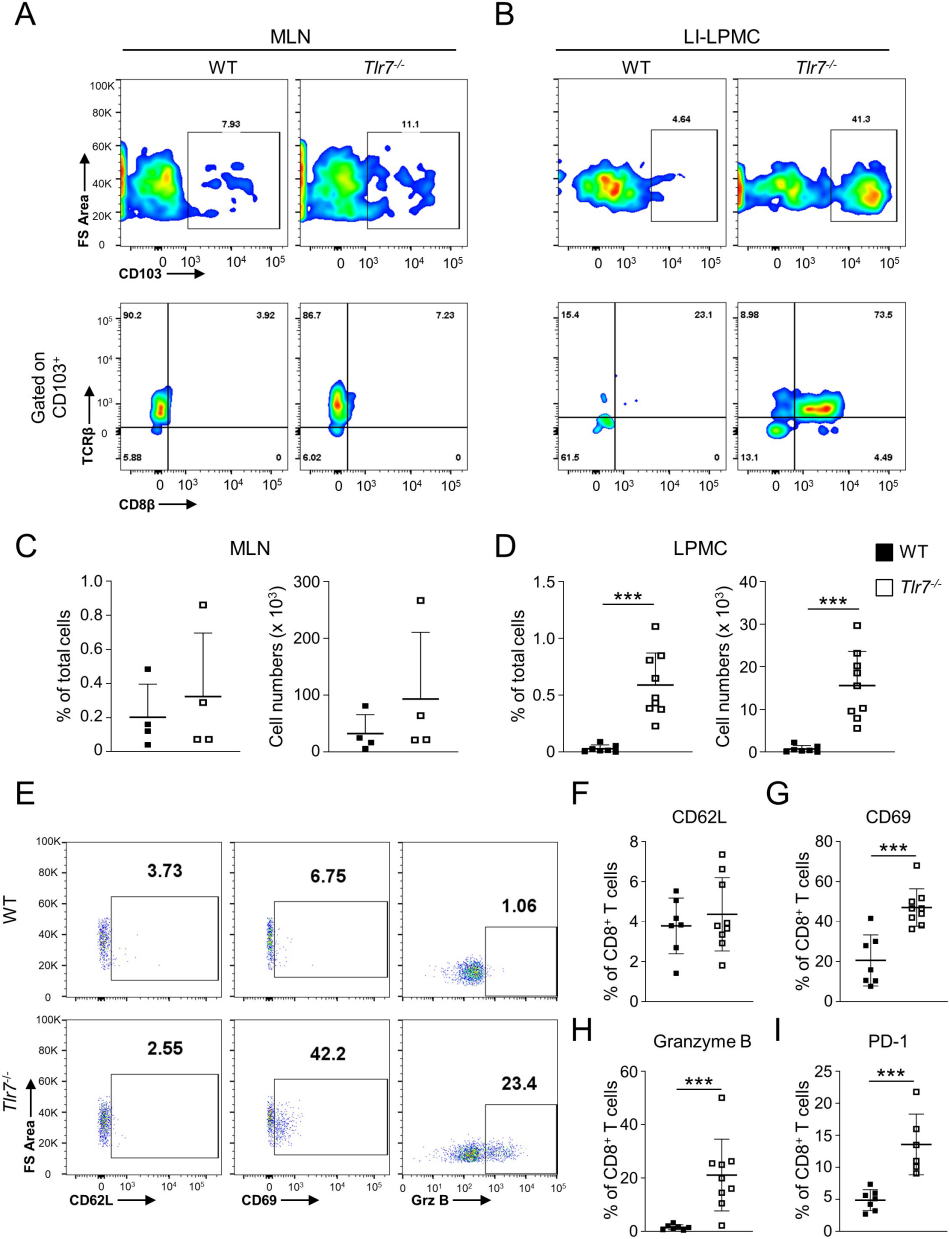
TLR7-deficiency leads to increased development of tissue-resident memory CD8^+^ T cells (T_RM_) in the intestine. WT and *Tlr7^-/-^* mice were analyzed under steady-state conditions. **(A, B)** Representative flow cytometry plots and **(C, D)** quantification of CD8^+^ T_RM_ cells as analyzed by flow cytometry and expressed as percentage of total cells (left panel) or total number of cells per organ (right panel). Cells isolate from the MLN **(A, C)**, or LI-LPMC **(B, D)** are gate on lymphocyte population. **(E)** Representative flow cytometry plots and **(F–I)** quantification of LI-LPMC CD8β^+^ CD62L^+^, CD8β^+^ CD69^+^, CD8β^+^ granzyme B^+^, CD8β^+^ PD-1^+^ cells as analyzed by flow cytometry and expressed as percentage of CD8β^+^ cells. Data represent means ± SD. Each symbol represents an individual mouse. ***p<0.005 as determined by Student’s *t-*test.

To assess the role of TLR7 signaling in maintaining intestinal homeostasis, we determined the total number mesenteric lymph nodes (MLN) and intestinal lamina propria mononuclear cells (LPMC) in WT and *Tlr7^-/-^* and observed no significant differences in LPMC but a significant increase of MLN cells in *Tlr7^-/-^* mice (**Supplementary Figure S1A**). The percentage of total LP CD8^+^ cells were increased in *Tlr7^-/-^* mice while LP DCs were not different between genotypes (**Supplementary Figure S1B**). Moreover, the percentage and total numbers of tissue-resident memory CD8^+^ T_RM_ cells (CD11c^+^MHCII^-^CD103^+^TCRβ^+^CD8β^+^) were significantly higher in LPMC of *Tlr7^-/-^* compared to WT mice, but similar in MLN, suggesting that the increase in this sub-population was limited to the LP (**Figures 1A–D**). To better characterize these CD8^+^ T_RM_ cells, we analyzed the percentage of CD62L^+^, CD69^+^, and Granzyme B^+^ CD8^+^ T_RM_ cells. We observed similar percentages of LP CD62L^+^ CD8^+^ T cells in *Tlr7^-/-^* mice but significantly higher percentages of CD69^+^ and Granzyme B^+^ CD8^+^ cells in *Tlr7^-/-^* compared to WT mice which correspond to the profile of tissue-resident memory CD8^+^ T cells (**Figures 1E–G**). Our data suggest that *Tlr7^-/-^* mice have an increased development of LP CD8^+^ T_RM_ cells compared to WT mice under steady-state conditions in the absence of histological inflammation (**Supplementary Figure S1C**).

### Intestinal CD8^+^ T_RM_ cells in Tlr7^-/-^ mice express high levels of PD-1

TLR7 is mainly expressed in pDCs and plays an important role in providing protection against viral infections. Next, we assessed if absence of TLR7 signaling and resulting impaired response to enteric viruses could lead to CD8^+^ T_RM_ cells exhaustion. LP CD8^+^ T_RM_ cells from *Tlr7^-/-^* mice had significantly higher percentages of the exhaustion marker PD-1 compared to WT mice (**Figure 1I**).

### Tlr7^-/-^ mice develop exacerbated acute colitis

**Figure 2:**
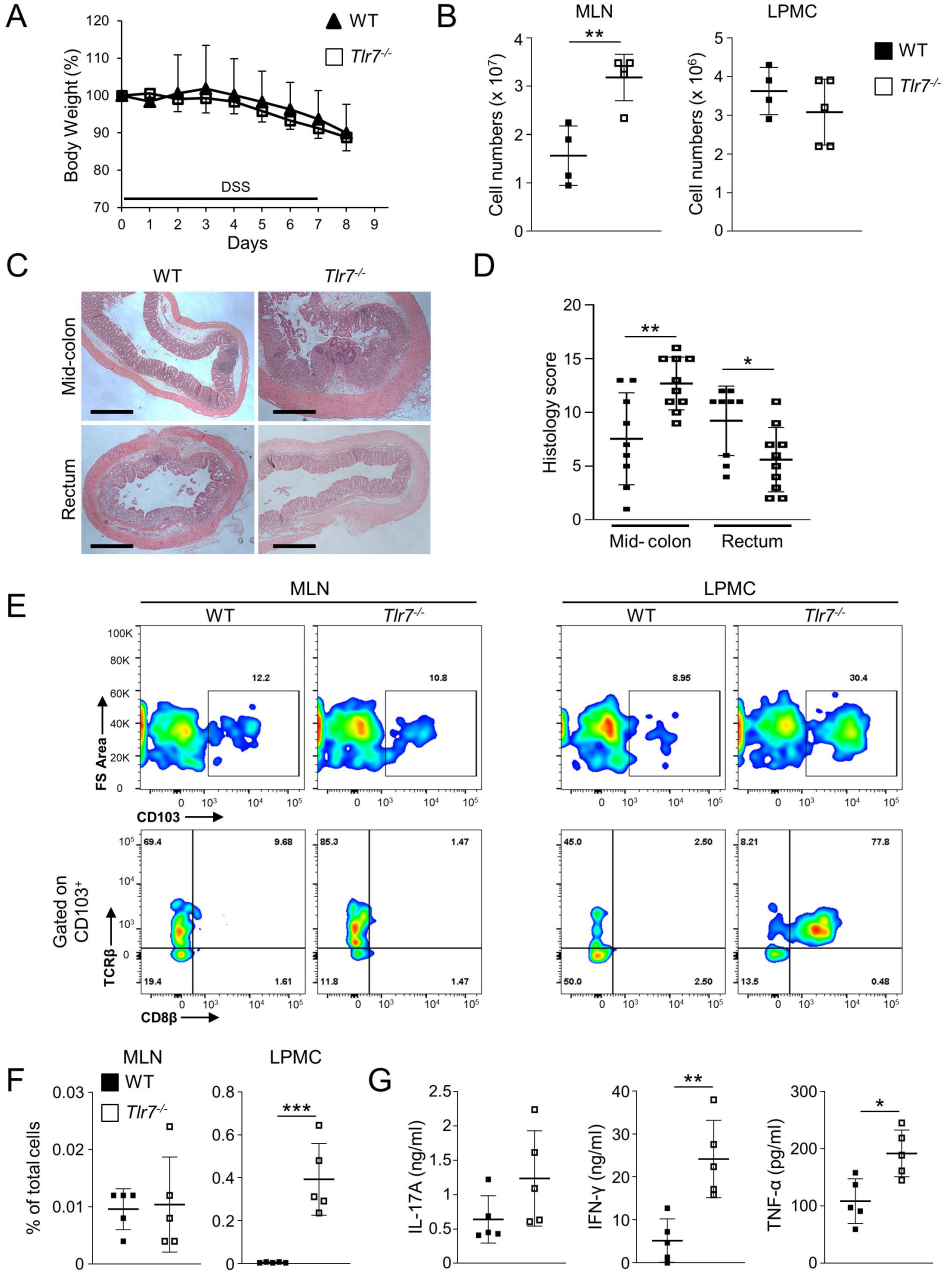
TLR7-deficiency worsens DSS-induced acute colitis. WT and *Tlr7^-/-^* mice underwent 7 days of DSS colitis. Mice were sacrificed at day 8 and assessed for intestinal inflammation. **(A)** Body weights as a percentage of the initial weight at day 1 of DSS. Representative experiment of three independent experiments. **(B)** Total numbers of mononuclear cells in MLN, and LP. **(C)** Representative H&E staining of intestinal sections and **(D)** Histology scores in different areas of the intestine. **(E)** Representative flow cytometry plots and **(F)** quantification of CD8^+^ T_RM_ cells as analyzed by flow cytometry and expressed as percentage of total cells. **(G)** LPMC were cultured with anti-CD3ϵ and anti-CD28 Abs. IFN-γ, TNF-α, and IL-17A secretion were measured by ELISA. Data represent means ± SD of one independent experiment out of at least three **(A, B, F, G)** independent experiments. Each symbol represents an individual mouse. *p<0.05, **p<0.01, ***p<0.005 as determined by Student’s *t-*test.

Based on our observation of an accumulation of LP T_RM_ cells, we hypothesized that *Tlr7^-/-^* mice are more susceptible to acute colitis. We administrated DSS in the drinking water for 6 days following by 1 day of regular drinking water to WT or *Tlr7^-/-^* mice. We did not observe any significant differences in the percentages of body weight loss between WT and *Tlr7^-/-^* mice after administration of DSS (**Figure 2A**). Total number of cells in MLN were significantly higher in *Tlr7^-/-^* compared to WT mice but were similar in LP (**Figure 2B**). *Tlr7^-/-^* mice had increased inflammation in the mid-colon area and decreased inflammation in the rectum compared to WT mice (**Figures 2C, D**). Similar to our observations during steady-state, the numbers of CD8^+^ T_RM_ were significantly higher in the LP of *Tlr7^-/-^* mice compared to WT mice but was similar in MLN in both genotypes (**Figures 2E, F**). Although the percentages of LP CD4^+^ T cells were significantly higher in *Tlr7^-/-^* compared to WT mice, proliferating CD4^+^ T cells and absolute numbers of LP CD4^+^ T cells and proliferating CD4^+^ T cells were similar between WT and *Tlr7^-/-^* mice (**Supplementary Figures S2A, B**). Next, we analyzed the secretion of T_H_1 and T_H_17 cytokines. *Tlr7^-/-^* mice had significantly higher secretion of IFN-γ and TNF-α in LP while IL-17A secretion was similar between *Tlr7^-/-^* and WT mice (**Figure 2G**).

### Tlr7^-/-^ mice develop exacerbated chronic colitis

**Figure 3:**
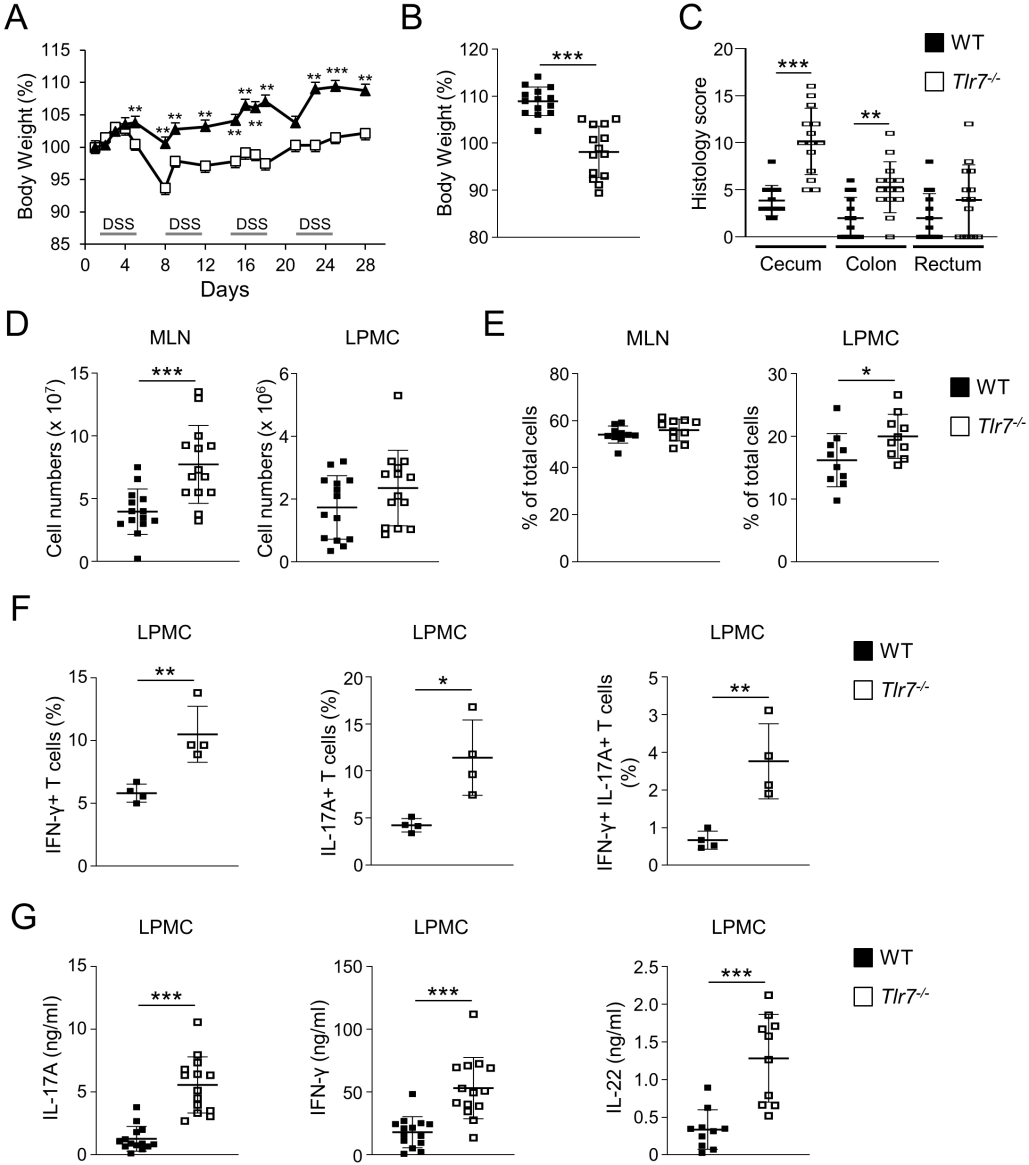
TLR7-deficiency worsens DSS-induced chronic colitis. WT and *Tlr7^-/-^* mice underwent four cycles of DSS colitis. Mice were sacrificed at day 29 and assessed for intestinal inflammation. **(A)** Body weights as a percentage of the initial weight at day 1 during the curse of colitis. Representative experiment of three independent experiments. **(B)** Body weights at the day of sacrifice. **(C)** Histology scores in different areas of the intestine. **(D)** Total numbers of mononuclear cells in MLN, and LP. **(E)** Quantification of CD4^+^ T cells in MLN, and LP as analyzed by flow cytometry and expressed as percentage of total cells. **(F)** Quantification of IFN-γ^+^, IL-17A^+^, and IFN-γ^+^IL-17A^+^ cells as analyzed by flow cytometry and expressed as percentage of CD4^+^ cells. **(G)** LPMC were cultured with anti-CD3ϵ and anti-CD28 Abs. IFN-γ, IL-17A, and IL-22 secretion were measured by ELISA. Data represent the mean ± SD. Each symbol represents an individual mouse. *p<0.05, **p<0.01, ***p<0.005 as determined by Student’s *t-*test.

To assess the role of TLR7 signaling in the development of chronic colitis we utilized the chronic DSS colitis model by administering four cycles of DSS drinking water to WT or *Tlr7^-/-^* mice. *Tlr7^-/-^* mice developed significantly more severe weight loss compared to WT mice starting during the first cycle of DSS treatment and continued throughout the entire experiment (**Figure 3A**). At the time of sacrifice *Tlr7^-/-^* mice had lost significantly more weight compared to WT mice (**Figure 3B**). We observed significantly higher histoscores characterized by more severe ulcerations, and cellular infiltration into the cecal and colonic LP in *Tlr7^-/-^* compared to WT mice (**Figure 3C**; **Supplementary Figure S3**). Total cell counts were significantly higher in MLN of *Tlr7^-/-^* compared to WT mice (**Figure 3D**). We did not observe significant differences in total cell counts for LP in *Tlr7^-/-^* compared to WT mice (**Figure 3D**). However, when we analyzed the cellular composition, we observed significant increases in the percentages of CD4^+^ T cells in LP in *Tlr7^-/-^* mice (**Figure 3E**) and increases of IFN-γ^+^, IL-17A^+^, and IFN-γ^+^ IL-17A^+^ double-positive CD4^+^ T cells (**Figure 3F**). We observed significant increases in the secretion of IL-17A, IFN-γ, and IL-22 from LPMC that were re-stimulated with anti-CD3/anti-CD28 (**Figure 3G**). Interestingly, we did not observe any differences in the secretion of these cytokines in MLN cells (data not shown). These data suggest that TLR7-deficiency leads to an exacerbation of localized intestinal T_H_1 and T_H_17 responses.

**Figure 4:**
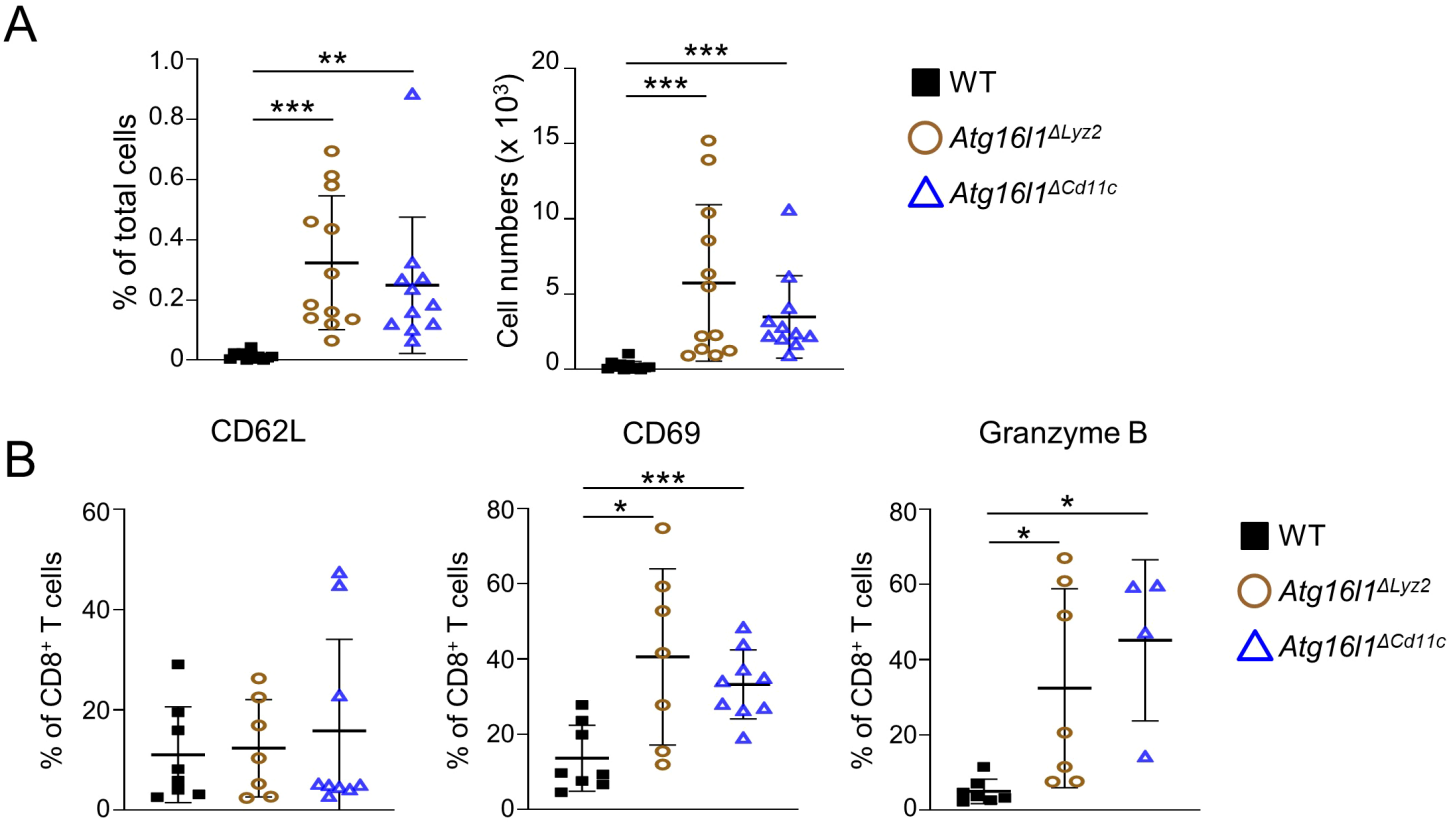
Myeloid- or dendritic cell-specific deletion of *Atg16l1* leads to increased development of tissue-resident memory CD8^+^ T_RM_ cells in the intestine. WT, *Atg16l1^ΔLyz2^*, and *Atg16l1^ΔCd11c^* mice were analyzed under steady-state conditions. **(A)** Quantification of LI-LP CD8^+^ T_RM_ cells as analyzed by flow cytometry and expressed as percentage of total cells (left panel) or total number of cells per organ (right panel) in the indicated mouse strains. **(B)** Quantification of LI-LP CD8β^+^ CD62L^+^, CD8β^+^ CD69^+^, CD8β^+^ granzyme B^+^ cells as analyzed by flow cytometry and expressed as percentage of CD8β^+^ cells. Data represent means ± SD. Each symbol represents an individual mouse. *p<0.05, **p<0.01, ***p<0.005 as determined by Student’s *t-*test.

### T cell intrinsic TLR7 signaling does not contribute to the development of chronic colitis

To determine if T cell intrinsic TLR7 signaling contributes to the development of chronic colitis, we transferred CD4^+^ CD45RB^hi^ naive T cells isolated from wild-type (WT) or *Tlr7^-/-^* mice into *Rag1^-/-^* mice and monitored for development of inflammatory disease. Mice that received *Tlr7^-/-^* CD4^+^ T cells displayed a similar degree of weight loss at the time of sacrifice than WT recipients (**Supplementary Figure S4A**). We did not observe any significant differences in the degree of intestinal inflammation in mice receiving WT or *Tlr7^-/-^* T cells (**Supplementary Figures S4B, C**). We observed a significant increase in LP cell numbers in mice receiving *Tlr7^-/-^* T cells while the cell numbers in MLN, and spleens of mice receiving WT or *Tlr7^-/-^* T cells were similar (**Supplementary Figure S4D**). Analysis of cellular composition revealed no significant differences in the percentages of CD4^+^ T cells in spleens but a significant increase of MLN cells in mice receiving *Tlr7^-/-^* T cells (**Supplementary Figure S4E**). However, we did observe any differences in the percentages of IL-17^+^IFN-γ^+^ CD4^+^ T cells in MLN or spleens of mice receiving WT or *Tlr7^-/-^* T cells (**Supplementary Figure S4F**). Next, we analyzed cytokine production upon TCR re-stimulation in LP and MLN. Although we did observe significantly increased numbers of LPMC we did not observe any differences in the secretion of IL-17A or IFN-γ in re-stimulated LPMC or MLN cells between mice receiving WT or *Tlr7^-/-^* T cells (**Supplementary Figures S4G, H**). We also did not observe any significant differences in the percentage of activated CD4^+^ T cells or percentage of CD4^+^ T cells expressing the gut-homing chemokine receptor CCR9 or the chemokine receptor CCR6 that identifies T_H_17 cells in LPMC or MLN from mice receiving WT or *Tlr7^-/-^* T cells (data not shown). These data suggest that TLR7 signaling in T cells does not contribute to the development of T cell-driven chronic colitis.

### Deletion of Atg16l1 in myeloid cells leads to increased development of CD8^+^ T_RM_ cells in the intestine

Chronic viral infections are associated with an increase in the numbers of CD8^+^ T_RM_ cells and a shift towards an exhausted phenotype of CD8^+^ T cells, which has been associated with defective pDC cytokine secretions (29, 50). We hypothesized that TLR7-deficiency leads to increased numbers of CD8^+^ T_RM_ cells due to defective anti-viral responses of pDC. To test our hypothesis, we used two conditional knock-out mouse strains with specific deletion of *Atg16l1* in myeloid cells (*Atg16l1^ΔLyz2^*) or DCs (*Atg16l1^ΔCd11c^*). At steady-state, *Atg16l1^ΔCd11c^* and *Atg16l1^ΔLyz2^* developed a similar phenotype than *Tlr7^-/-^* mice with increased percentage and total number of CD8^+^ T_RM_ cells in the LP (**Figure 4A**). CD8^+^ T_RM_ cells in *Atg16l1^ΔCd11c^* and *Atg16l1^ΔLyz2^* mice had similar percentages of CD62L^+^ but higher percentages of CD69^+^ and granzyme B^+^ cells compared to WT mice (**Figure 4B**).

### PDC and bone marrow-derived macrophages from Atg16l1^ΔCd11c^ mice respond to TLR7 agonist Imiquimod

**Figure 5:**
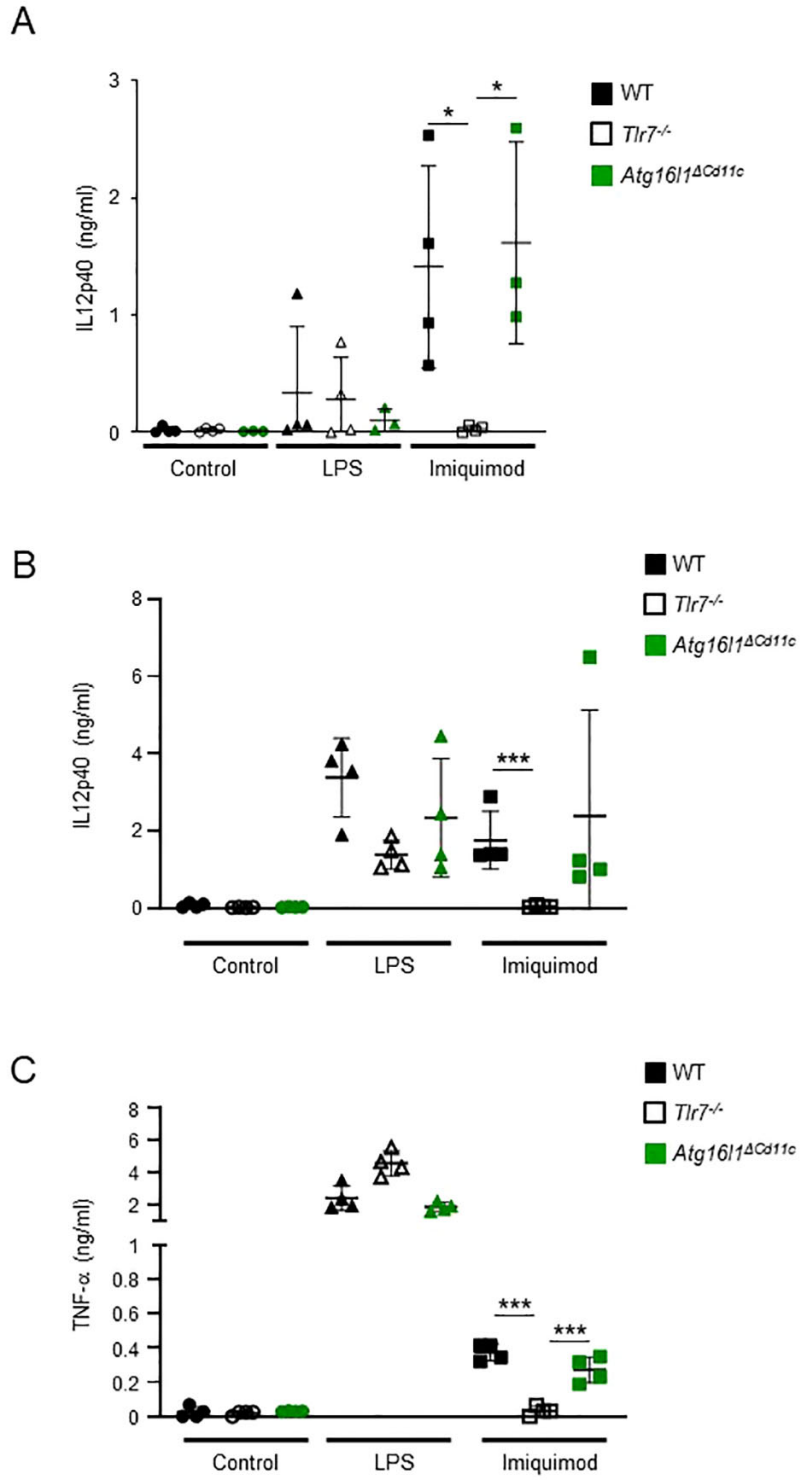
Bone-marrow derived pDCs and macrophages from *Atg16l1^ΔCd11c^* mice respond to Imiquimod. **(A)** Bone-marrow derived pDCs from WT, *Tlr7^-/-^*, and *Atg16l1^ΔCd11c^* mice were cultured *in vitro* and stimulated with or without LPS or Imiquimod for 24 h. IL12p40 secretion in supernatants were measured by ELISA. **(B, C)** Bone-marrow derived macrophages from WT, *Tlr7^-/-^*, and *Atg16l1^ΔCd11c^* mice were cultured *in vitro* and stimulated with or without LPS or Imiquimod for 24 h. IL12p40 **(B)** and TNF-α **(C)** secretion in supernatants were measured by ELISA. Data represent means ± SD. N=4. *p<0.05, ***p<0.005 as determined by Student’s *t-*test.

In order to determine if direct activation of TLR7 using the agonist Imiquimod could bypass the requirement for ATG16L1 in myeloid cells, we cultured bone marrow-derived pDCs isolated from WT, *Tlr7^-/-^*, *Atg16l1^ΔCd11c^*, and *Atg16l1^ΔLyz2^* mice and stimulated them with LPS or Imiquimod *in vitro.* pDCs isolated from all genotypes had increased secretion of IL12p40 after stimulation with LPS, demonstrating their capacity to respond to a TLR4 ligand (**Figure 5A**; **Supplementary Figure S5**). Stimulation with Imiquimod increased secretion of IL12p40 in pDCs isolated from WT, *Atg16l1^ΔCd11c^*, and *Atg16l1^ΔLyz2^* mice but not from *Tlr7^-/-^* mice as expected (**Figure 5A**; **Supplementary Figure S5**). Similarly, Imiquimod increased secretion of IL12p40 and TNF-α in bone marrow-derived macrophages isolated from WT and *Atg16l1^ΔCd11c^* mice but not from *Tlr7^-/-^* mice as expected (**Figures 5B, C**). These data demonstrates that stimulation with Imiquimod *in vitro* could bypass the defect in ATG16L1 and activate TLR7 signaling in pDCs and macrophages.

### Deletion of Atg16l1 in myeloid cells worsens acute colitis that could be attenuated by Imiquimod treatment

**Figure 6:**
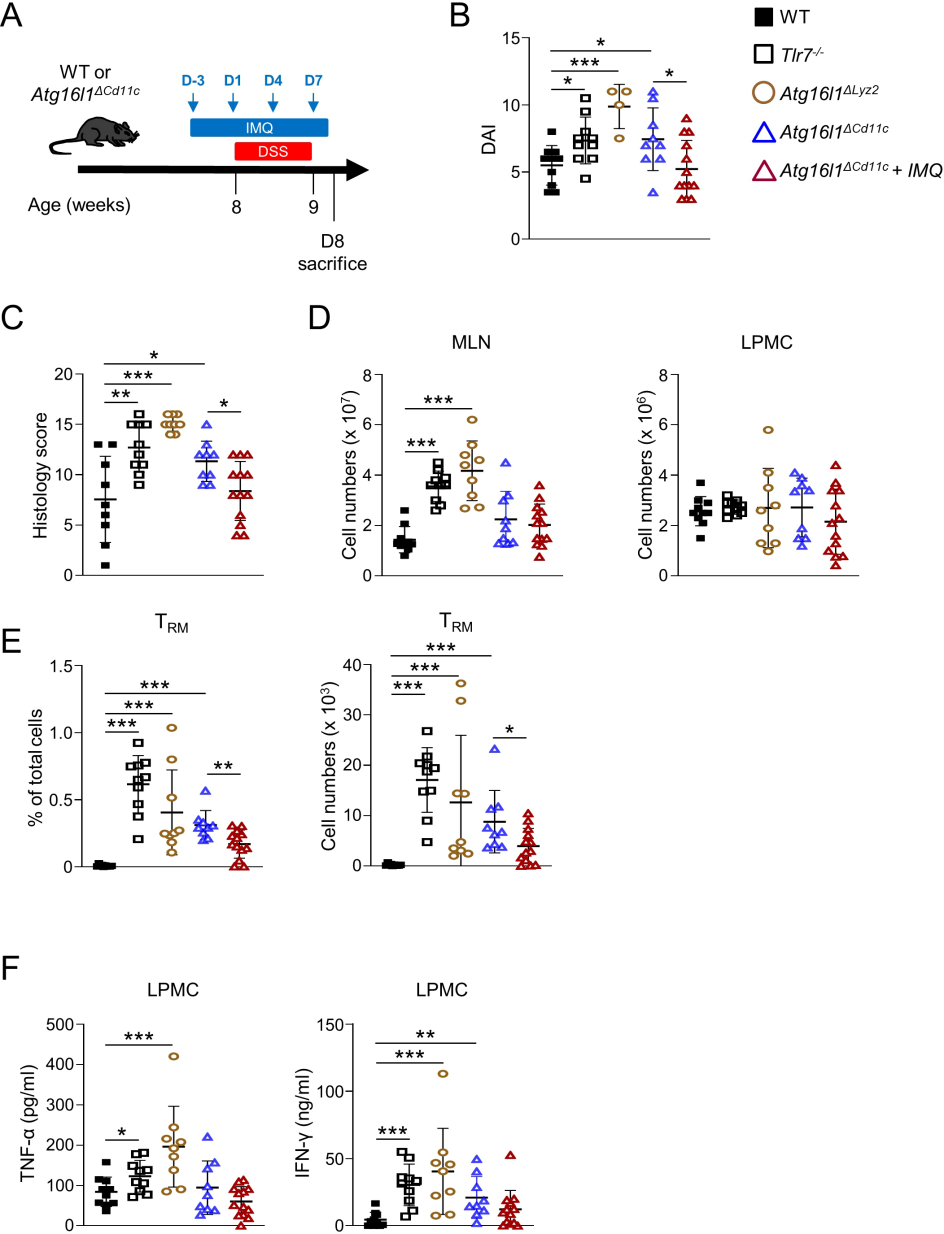
Myeloid-specific deletion of *Atg16l1* worsens DSS-induced acute colitis that is attenuated by imiquimod treatment. Control (WT), *Atg16l1^ΔLyz2^*, and *Atg16l1^ΔCd11c^* mice underwent 7 days of DSS colitis. Mice were sacrificed at day 8 and assessed for intestinal inflammation. A group of *Atg16l1^ΔCd11c^* mice received a preventive treatment of imiquimod (i.p.) on day -3, 1, 4, 7 during the DSS administration. **(A)** Schematic of the experimental design. **(B)** DAI scores. **(C)** Histology scores of mid-colons. **(D)** Total numbers of mononuclear cells in MLN (left), and LP (right). **(E)** Quantification of LI-LP CD8^+^ T_RM_ cells as analyzed by flow cytometry and expressed as percentage of total cells (left panel) or total number of cells per organ (right panel). **(F)** LPMC were cultured with anti-CD3ϵ and anti-CD28 Abs. IFN-γ and TNF-α secretion were measured by ELISA. Each symbol represents an individual mouse. Data represent means ± SD. Representative experiment of at least two independent experiments. *p<0.05, **p<0.01, ***p<0.005 as determined by Student’s *t-*test.

To further investigate the relationship between TLR7 and ATG16L1 signaling, we administrated DSS in the drinking water of WT, *Tlr7^-/-^*, *Atg16l1^ΔLyz2^*, and *Atg16l1^ΔCd11c^* mice and treated *Atg16l1^ΔCd11c^* mice with Imiquimod for 10 days (**Figure 6A**). *Tlr7^-/-^*, *Atg16l1^ΔLyz2^*, and *Atg16l1^ΔCd11c^* mice were more susceptible to DSS as apparent by increased disease activity index and histoscores compared to WT mice (**Figures 6B, C**; **Supplementary Figure S6A**). *Tlr7^-/-^* and *Atg16l1^ΔLyz2^* mice had increased total cell numbers in MLN compared to WT mice, but similar total cell numbers in the LP (**Figure 6D**). Percentage and total number of LP CD8^+^ T_RM_ cells and secretion of IFN-γ and TNF-α were increased in *Tlr7^-/-^*, *Atg16l1^ΔLyz2^*, and *Atg16l1^ΔCd11c^* compared to WT mice (**Figures 6E, F**). In contrast, colonic *Ifnb1* mRNA expression was similar between WT, *Tlr7^-/-^*, and *Atg16l1^ΔCd11c^* but significantly reduced in *Atg16l1^ΔLyz2^* compared to WT mice (**Supplementary Figure S6B**). *Atg16l1^ΔCd11c^* mice treated with Imiquimod showed attenuated DSS-induced inflammation compared to untreated *Atg16l1^ΔCd11c^* mice with significantly decreased disease activity index, histoscores, and percentages and total numbers of LP CD8^+^ T_RM_ cells (**Figures 6B, C, E**). Secretion of IFN-γ and TNF-α were decreased but did not reach statistical significance (**Figure 6F**). Our data demonstrate that ATG16L1-deficiency in DCs promotes the development of LP CD8^+^ T_RM_ cells and increases susceptibility to colitis which can be attenuated by stimulation of the TLR7 signaling pathway.

## Discussion

We investigate the role of TLR7 signaling pathway and its convergence with ATG16L1 pathway in intestinal homeostasis and inflammation. Our findings reveal that TLR7 protects from the development of colonic inflammation in both acute and chronic colitis. The absence of TLR7 leads to increased numbers of LP CD8^+^ T_RM_ but not MLN CD8^+^ T_RM_ cells suggesting that the effects of TLR7-deficiency on CD8^+^ T_RM_ cells were mainly confined to the mucosa.

The role of CD8^+^ T cells in IBD has been controversial, with studies in UC and CD patients showing their role in promoting inflammation while some murine models showed a protective role against colitis (41, 51–58). This discrepancy may be partially explained by the peripheral blood vs mucosal tissue origin of the CD8^+^ T cells investigated, as well as by a substantial heterogeneity within the CD8^+^ T cell population (40, 59, 60). Here, we observed an increase in CD8^+^ T_RM_ cells in the LP but not in the MLN of *Tlr7^-/-^* mice, suggesting a unique milieu at the mucosal site that promotes their expansion. This finding aligns well with studies showing that eukaryotic viral infections predominantly induce immune responses at the sites of virus entry and replication (29). Single-cell RNA sequencing of biopsies from healthy individuals and UC patients revealed a significant heterogeneity among CD8^+^ T_RM_ cells (40, 42). This analysis revealed four distinct transcriptional states of differentiation in CD8^+^ T_RM_ cells across both healthy and diseased tissues, including the expansion of a particularly inflammatory CD8^+^ T_RM_ subset, characterized by high expression of granzyme A, B, H, K, and M, IFN-γ, KLRG1, the transcription factors Eomes and ZEB2, and the metabolic regulator FABP5 in affected colonic tissue from UC patients (40). The increase of intestinal inflammation in *Tlr7^-/-^* mice correlated with an increase in granzyme B^+^ CD8^+^ T_RM_ cells, IFN-γ, and TNF-α secretion during DSS colitis and suggest that CD8^+^ T_RM_ cells contribute to the increased susceptibility seen in *Tlr7^-/-^* mice. Indeed, depletion of CD8^+^ T_RM_ cells in the T cell transfer and TNBS models of colitis prevented the development of colonic inflammation (36). Furthermore, animal studies demonstrated that IFN-γ-producing cytotoxic CD8^+^ T cells could promote development of colitis through secretion of IFN-γ and TNF-α and their detrimental effects on intestinal epithelial cells (61, 62). Increased TNF-α secretion observed in *Tlr7^-/-^*, *Atg16l1* ^Cd11cCre^, and *Atg16l1* ^Lyz2^ mice may contribute to the susceptibility to DSS-induced colitis via its detrimental effects on intestinal epithelial cells. Further investigation into these mechanisms is warranted in future studies.

TLR7 is primarily expressed in pDC, but also in various other immune cells such as macrophages, T cells, and B cells. Our data exclude an intrinsic role of TLR7 signaling in CD4^+^ effector cells. These observations suggest that the increase in CD8^+^ T_RM_ cells in TLR7-deficient mice may be due to a defect in myeloid cell function. Type I IFN attenuation during chronic viral infections contribute to T cell exhaustion and the persistence of the virus (50, 63–65). Absence of pDCs in mice diminishes their ability to effectively recognize and respond to acute and chronic viral infections (66, 67). CD8^+^ T cell responses differ between different norovirus strains causing either acute or persistent infections (14, 68). Comprehensive profiling of immune responses to eukaryotic enteric viruses reveals that infection with the persistent MNV strain CR6 induces an increase in granzyme B^+^ CD8^+^ and IFN-γ^+^ CD8^+^ T cells in the colonic LP, whereas the non-persistent MNV strain CW3 does not (29). In our study, we hypothesized that increased in CD8^+^ T_RM_ cells was a result of defective anti-viral responses in myeloid cells. Upon entry of viruses into cells ATG16L1-dependent autophagosomes engulf viruses, degrade, and transport viral components to endosomes, where viral ssRNA will be recognized by TLR7, activating its downstream signaling pathway. Further studies demonstrating a direct involvement of MNV infections in the increase in CD8^+^ T_RM_ cells in *Tlr7^-/-^* and mice with ATG16L1-deficieny in myeloid cells or DCs are warranted.

As previously mentioned, association between the risk variant ATG16L1 T300A, persistent norovirus infection and increase susceptibility to colitis was demonstrated (13, 14). Here, we aimed to explore whether the susceptibility to norovirus infection was partially due to its incapacity to activate TLR7 signaling in myeloid cells. ATG16L1-deficieny in myeloid cells or DCs resulted in a similar phenotype than *Tlr7^-/-^* mice, with increased susceptibility to DSS colitis, increased disease activity index, histoscores, and increased secretion of IFN-γ and TNF-α. Those data suggested a convergence of TLR7 and ATG16L1 signaling pathways in myeloid cells and demonstrated that either TLR7-deficieny or ATG16L1 defects in myeloid cells are sufficient to drive an expansion of CD8^+^ T_RM_ cells observed in *Tlr7^-/-^*, *Atg16l1* ^Cd11cCre^, and *Atg16l1* ^Lyz2^ mice. Subsequently, we explored whether treatment with the TLR7 agonist Imiquimod could bypass the defective autophagy-dependent activation of TLR7 signaling in *Atg16l1* ^Cd11cCre^ mice *in vivo*. We treated *Atg16l1^ΔCd11c^* mice with Imiquimod and observed a significant attenuation of mucosal inflammation. TLR7-independent effects of Imiquimod have been suggested in no-immune cells that don’t express TLR7 via its interference with adenosine receptor signaling mediated by adenylyl cyclase (69, 70). It remains to be elucidated if TLR7-independent effects of Imiquimod contribute to the attenuation of mucosal inflammation in *Atg16l1^ΔCd11c^* mice with Imiquimod.

Future studies investigating whether activation of the TLR7 signaling pathway might be beneficial for CD patients with *ATG16L1* risk variants are warranted.

In conclusion, we demonstrated that relation between ATG16L1 and TLR7 plays an important role in maintaining immune response to intestinal viruses and protects against colitis.

## Data Availability

The original contributions presented in the study are included in the article/**Supplementary Material**. Further inquiries can be directed to the corresponding author.
