# Supplementary material for: Toll-like receptor 7 protects against intestinal inflammation and restricts the development of colonic tissue-resident memory CD8+ T cells
Source: Front Immunol. 2024 Oct 11;15:1465175. doi: 10.3389/fimmu.2024.1465175 (PMC11502343; doi:10.3389/fimmu.2024.1465175)
Supplement: Supplementary file 1 [file DataSheet1.pdf]

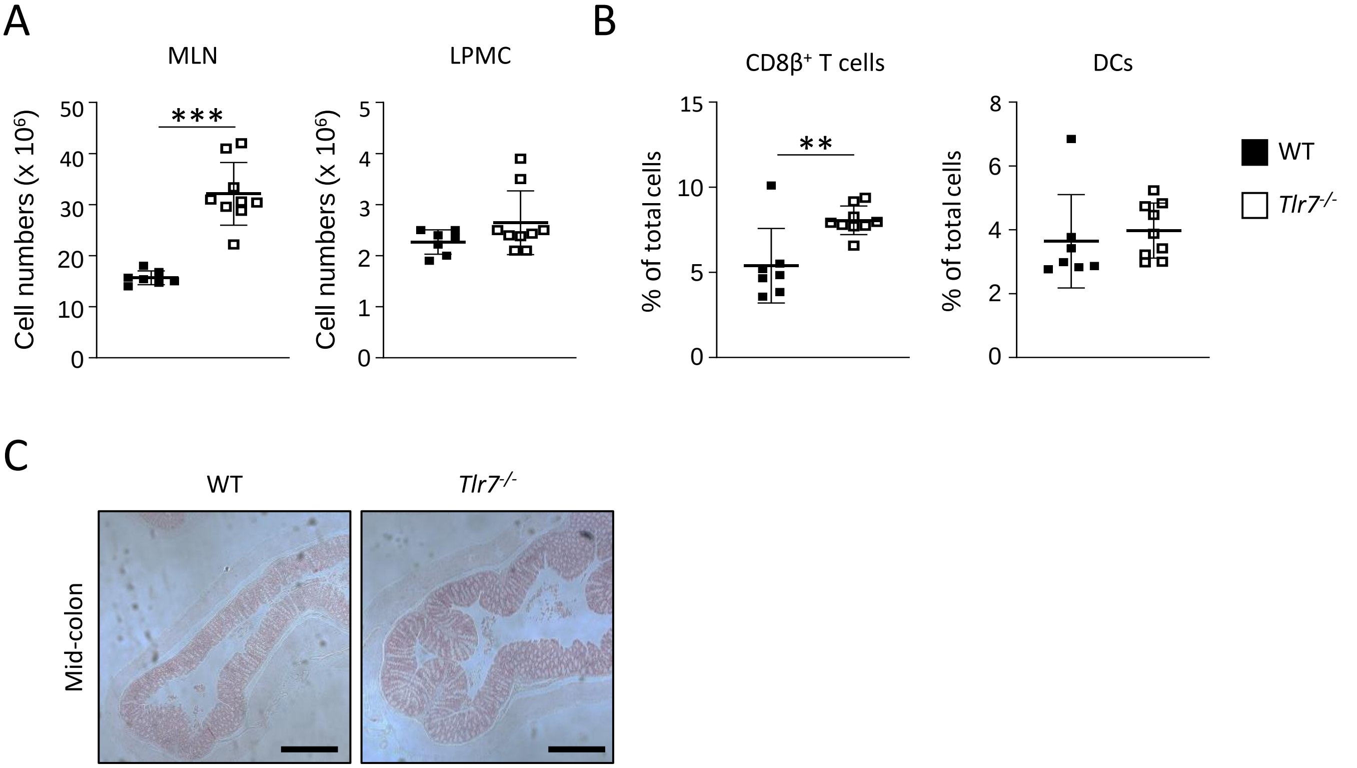

Supplemental Figure 1. TLR7-deficient mice do not development spontaneous intestinal inflammation. WT and *Tlr7*$^{-/-}$ mice at steady-state. **(A)** Total numbers of mononuclear cells in MLN (left), and LP (right). **(B)** Quantification of LP CD8β^{+} cells (left panel) and LP MHCII^{+}CD11c^{+} DCs (right panel) as analyzed by flow cytometry and expressed as percentage of total cells. **(C)** Representative H&E stainings of mid-colon sections are shown. Data represent means ± SD. **, *p*<0.01, ***, *p*<0.005 as determined by Student's *t*-test.

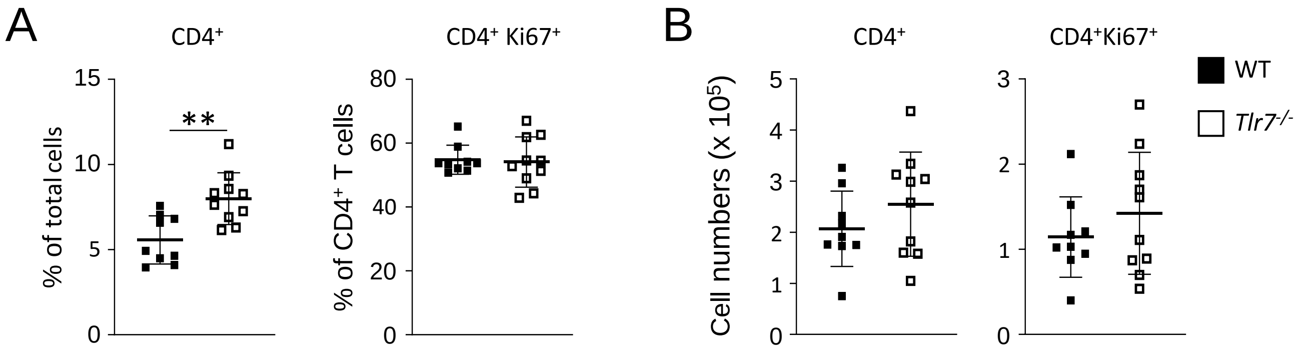

Supplemental Figure 2. TLR7-deficiency worsens DSS-induced acute colitis without changing the total numbers of CD4⁺ T cells. WT and *Tlr7*$^{-/-}$ mice underwent 7 days of DSS colitis. Mice were sacrificed at day 8 and assessed for intestinal inflammation. **(A)** Percentages of LP CD4^{+} T cells (left panel) and percentages of CD4^{+} Ki67^{+} T cells (right panel). **(B)** Total cell numbers of LP CD4^{+} T cells (left panel) and of CD4^{+} Ki67^{+} T cells (right panel). Each symbol represents an individual mouse. **, *p*<0.01 as determined by Student's *t*-test.

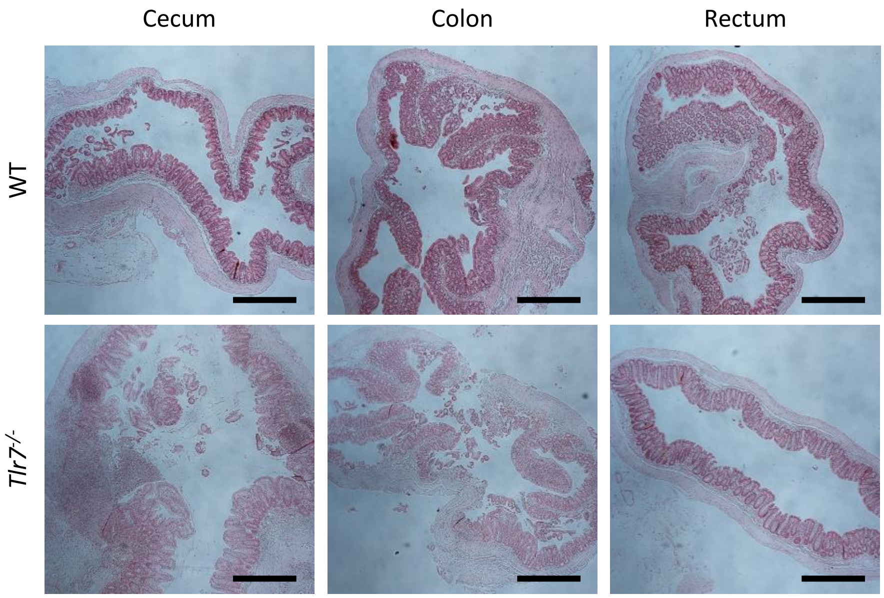

Supplemental Figure 3. TLR7-deficiency worsens cecal inflammation in chronic DSS-induced colitis. WT and *Tlr7*$^{-/-}$ mice underwent four cycles of DSS colitis. Mice were sacrificed at day 29 and assessed for intestinal inflammation. Representative H&E stainings of intestinal sections are shown.

Supplemental Figure 4

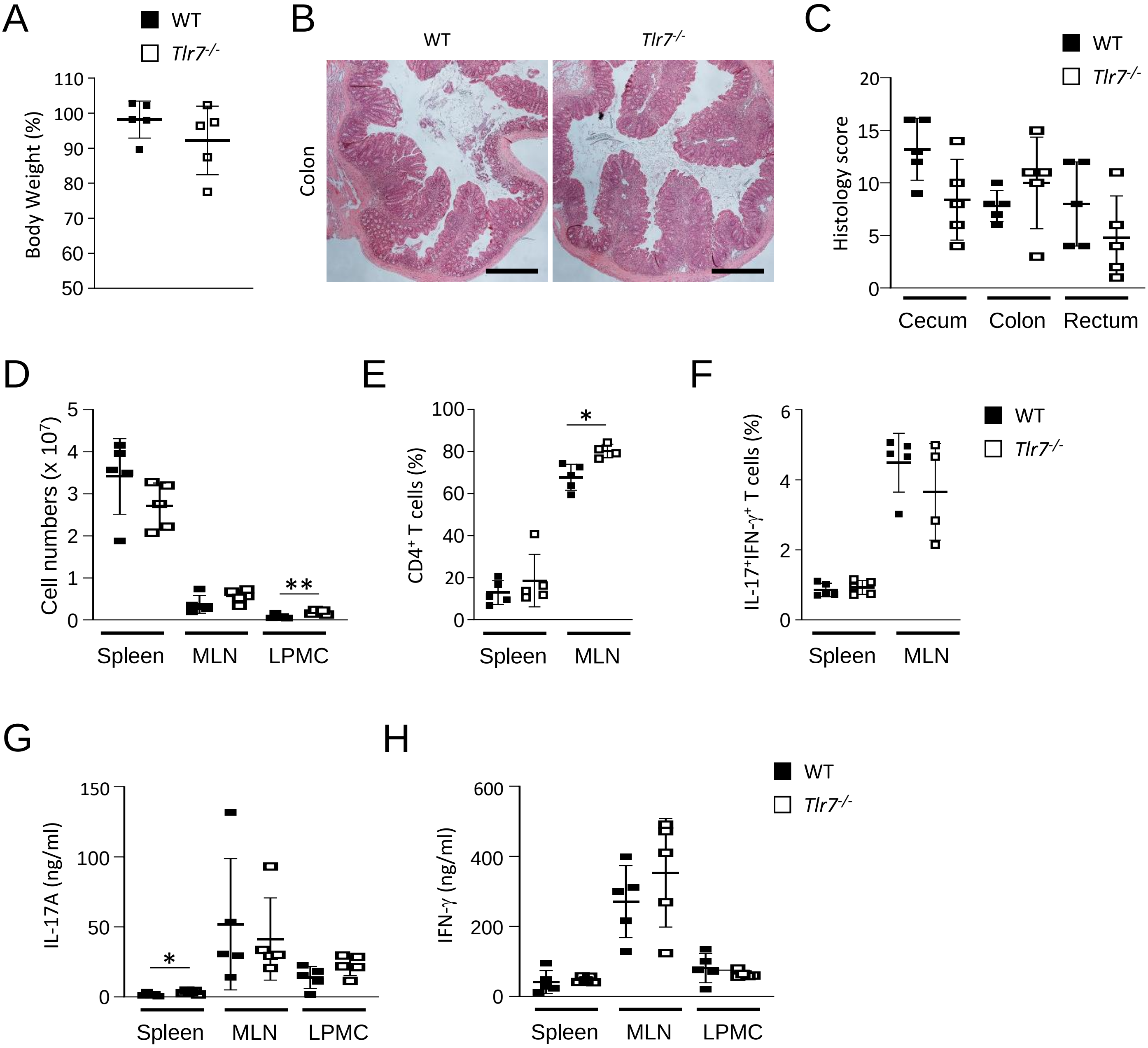

Supplemental Figure 4. TLR7-deficient T cells develop similar intestinal inflammation than WT T cells. *Rag1*$^{-/-}$ mice were transferred with WT or *Tlr7*$^{-/-}$ $CD4^{+}$ $CD45RB^{hi}$ T cells. Mice were sacrificed 6 weeks after T cell transfer and assessed for intestinal inflammation. **(A)** Body weights at the day of sacrifice. **(B)** Representative H&E staining of colon sections. **(C)** Histology scores in different areas of the intestine. **(D)** Total numbers of cells in Spleen, MLN, and LPMC. **(E)** Quantification of $CD4^{+}$ cells as analyzed by flow cytometry and expressed as percentage of total cells. **(F)** IFN-γ^{+}IL-$17A^{+}$ cells as analyzed by flow cytometry and expressed as percentage of $CD4^{+}$ cells. **(G)** Mononuclear cells from Spleens, MLN, and LP were cultured with anti-CD3e and anti-CD28 Abs. IFN-γ, and IL-17A secretion were measured by ELISA. Data represent the means ± SD. One representative experiment out of two independent experiments is shown. Statistical significance was determined by Student's *t*-test. *, $p<0.05$.

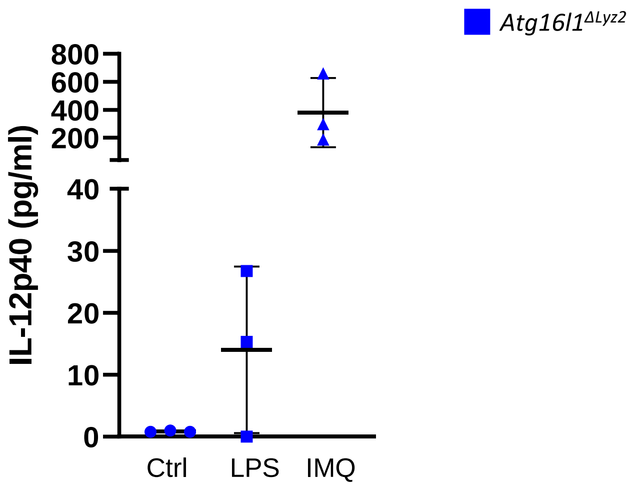

Supplemental Figure 5. Bone-marrow derived pDCs from *Atg16l1*$^{\Delta Lyz2}$ mice respond to Imiquimod. Bone-marrow derived pDCs from *Atg16l1*$^{\Delta Lyz2}$ mice were cultured *in vitro* and stimulated with or without LPS or Imiquimod for 24 h. IL12p40 secretion in supernatants were measured by ELISA. Data represent means ± SD. N=3.

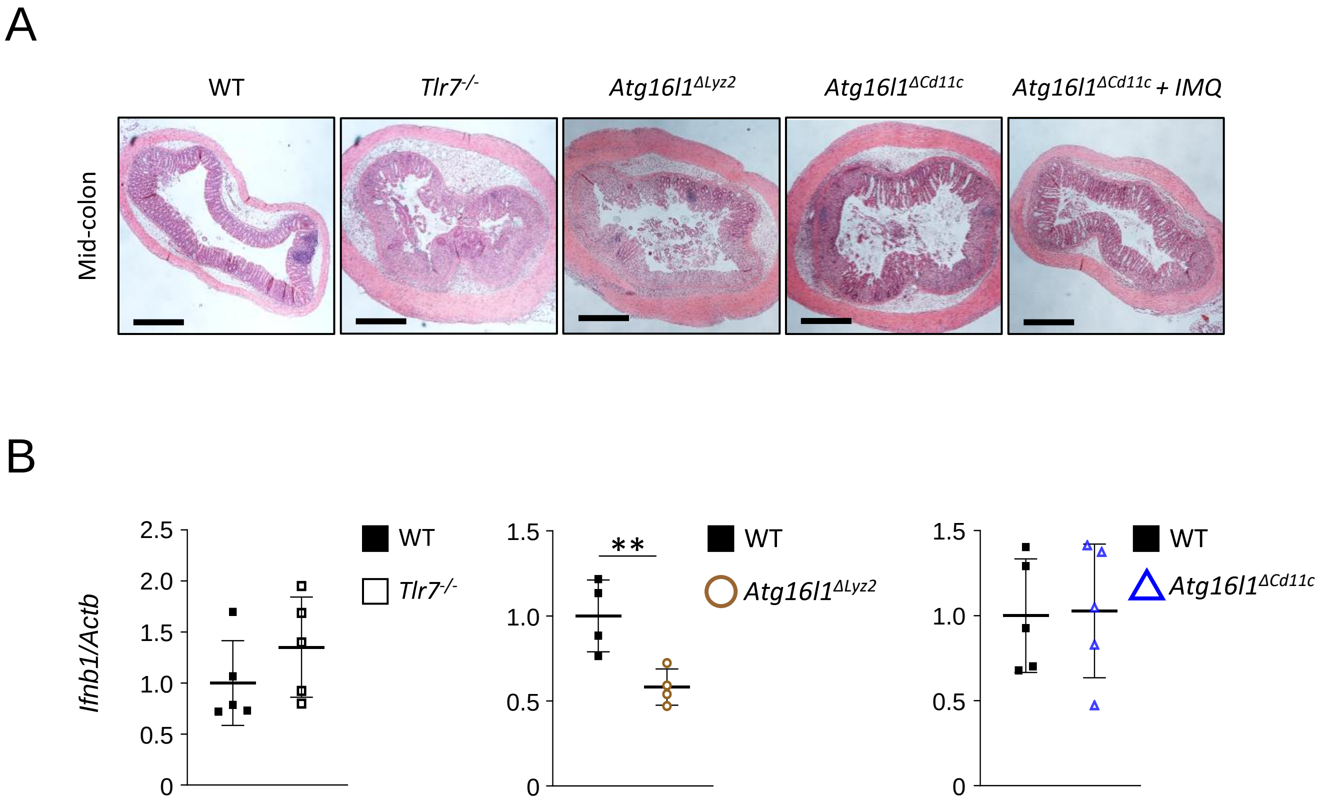

Supplemental Figure 6. Myeloid-specific deletion of *Atg16l1* worsens DSS-induced acute colitis that is attenuated by imiquimod treatment. Control (WT), *Tlr7*$^{-/-}$, *Atg16l1*$^{\Delta Lyz2}$, and *Atg16l1*$^{\Delta Cd11c}$ mice underwent 7 days of DSS colitis. Mice were sacrificed at day 8 and assessed for intestinal inflammation. *Atg16l1*$^{\Delta Cd11c}$ mice received imiquimod on days -3, 1, 4, and 7 of DSS administration. **(A)** Representative H&E stainings of mid-colon sections are shown. **(B)** mRNA expression of *Ifnb1* in the mid-colon of WT, *Tlr7*$^{-/-}$, *Atg16l1*$^{\Delta Lyz2}$, and *Atg16l1*$^{\Delta Cd11c}$ mice on day 8 of DSS administration measured by qPCR. All data were normalized to expression of *Actb* and represented as fold changes compared to WT mice (n = 4-5/group). Statistical significance was determined by Student's *t*-test. **, p<0.01.

Supplemental Figure 7

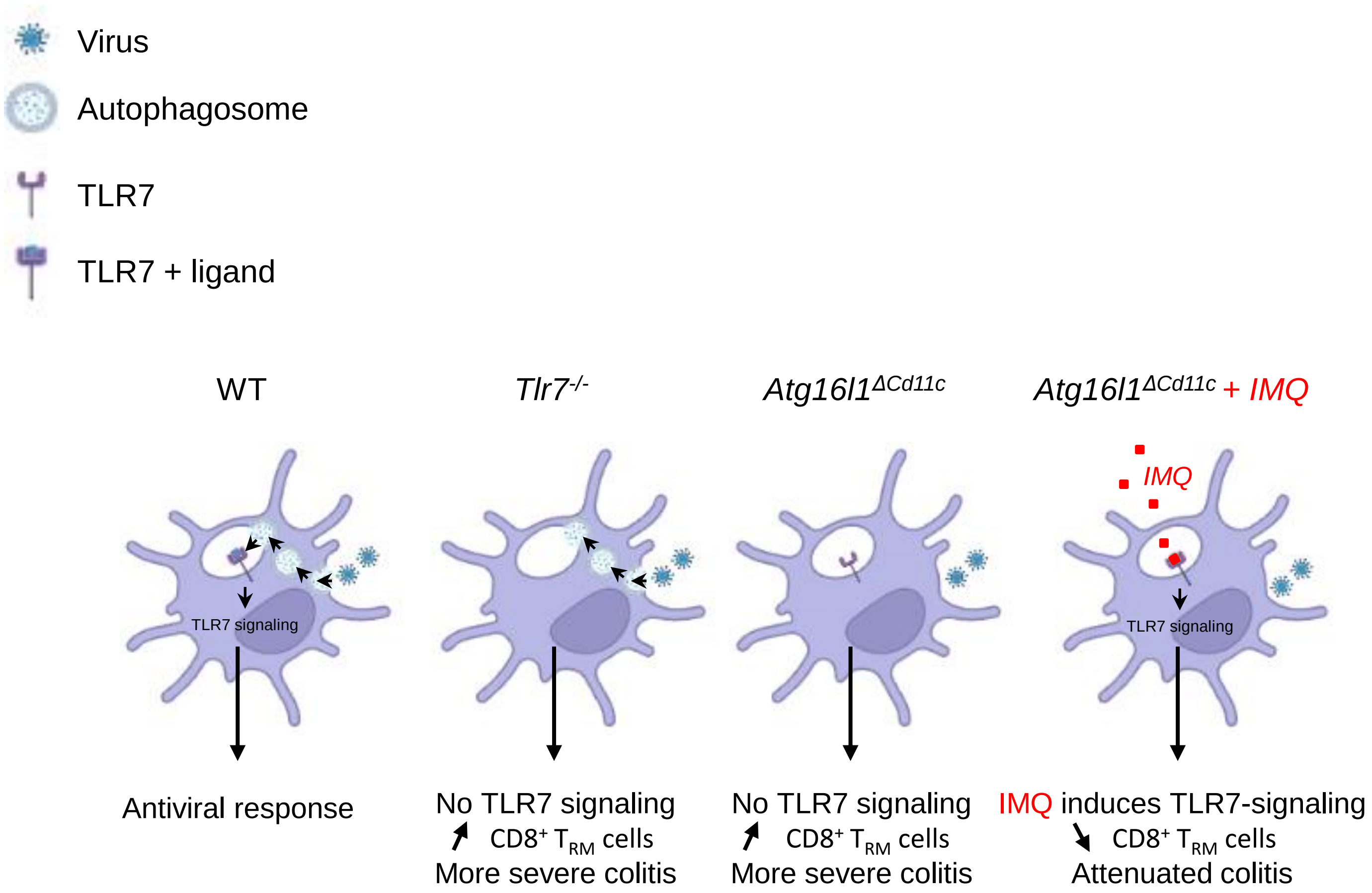

Supplemental Figure 7. Proposed mechanism of TLR7 and ATG16L1 interaction. In WT mice, ssRNA viruses are internalized by myeloid cells and delivered to endosomes through ATG16L1-dependent autophagosomes. In the endosomes ssRNA viruses bind TLR7 and activate it's signaling pathway. Activation of TLR7 triggers an antiviral response. In *Tlr7$^{-/-}$* mice, ssRNA are internalized but can not trigger an antiviral response due to the absence of TLR7. The absence of TLR7 signaling leads to an increase of CD8^{+} T_{RM} cells and increases the susceptibility to DSS-induced colitis. In *Atg16l1$^{\Delta Cd11c}$* mice, ssRNA viruses are not delivered to endosomes due to the impaired autophagosome formation due to the absence of ATG16L1. Consequently, TLR7 signaling pathways are not activated, resulting in an increase of CD8^{+} T_{RM} cells, and increased susceptibility to DSS-induced colitis. However, stimulation with IMQ, an TLR7 agonist, in *Atg16l1$^{\Delta Cd11c}$* mice, bypasses the defect of virus internalization and delivery to endosomes by directly activating TLR7. This leads to an antiviral response, reduction of CD8^{+} T_{RM} cells, and attenuation of DSS-induced colitis. This figure was partially created with BioRender.
